# Two recessive mutations in *FGF5* are associated with the long-hair phenotype in donkeys

**DOI:** 10.1186/s12711-014-0065-5

**Published:** 2014-09-25

**Authors:** Romain Legrand, Laurent Tiret, Marie Abitbol

**Affiliations:** UMR955 INRA-ENVA de Génétique Fonctionnelle et Médicale, Université Paris-Est, Institut National de la Recherche Agronomique, Ecole nationale vétérinaire d’Alfort, F-94700 Maisons-Alfort, France

## Abstract

**Background:**

Seven donkey breeds are recognized by the French studbook. Individuals from the Pyrenean, Provence, Berry Black, Normand, Cotentin and Bourbonnais breeds are characterized by a short coat, while those from the Poitou breed (Baudet du Poitou) are characterized by a long-hair phenotype. We hypothesized that loss-of-function mutations in the *FGF5* (*fibroblast growth factor 5*) gene, which are associated with a long-hair phenotype in several mammalian species, may account for the special coat feature of Poitou donkeys. To the best of our knowledge, mutations in *FGF5* have never been described in *Equidae*.

**Methods:**

We sequenced the *FGF5* gene from 35 long-haired Poitou donkeys, as well as from a panel of 67 short-haired donkeys from the six other French breeds and 131 short-haired ponies and horses.

**Results:**

We identified a recessive c.433_434delAT frameshift deletion in *FGF5*, present in Poitou and three other donkey breeds and a recessive nonsense c.245G > A substitution, present in Poitou and four other donkey breeds. The frameshift deletion was associated with the long-hair phenotype in Poitou donkeys when present in two copies (n = 31) or combined with the nonsense mutation (n = 4). The frameshift deletion led to a stop codon at position 159 whereas the nonsense mutation led to a stop codon at position 82 in the FGF5 protein. In silico, the two truncated FGF5 proteins were predicted to lack the critical β strands involved in the interaction between FGF5 and its receptor, a mandatory step to inhibit hair growth.

**Conclusions:**

Our results highlight the allelic heterogeneity of the long-hair phenotype in donkeys and enlarge the panel of recessive *FGF5* loss-of-function alleles described in mammals. Thanks to the DNA test developed in this study, breeders of non-Poitou breeds will have the opportunity to identify long-hair carriers in their breeding stocks.

**Electronic supplementary material:**

The online version of this article (doi:10.1186/s12711-014-0065-5) contains supplementary material, which is available to authorized users.

## Background

The hair growth cycle in mammals is composed of three phases: anagen, when follicles grow and hair is synthetized; catagen, when follicles regress; and telogen, when follicles are inactive [1]. Hair growth is regulated by several cytokines and growth factors, including members from the fibroblast growth factors family (FGF) and their receptors. Eighteen mammalian FGF have been described that exhibit a variety of activities, among which paracrine factors that are involved in tissue patterning and organogenesis during embryogenesis and endocrine factors that are involved in metabolism homeostasis during adulthood [2]. Among the members of the FGF family, FGF5 was first described as a human oncogene [3] and was later shown to be involved in the dual-mode regulation of the hair growth cycle [4,5]. In rats and mice, alternative splicing of *Fgf5* transcripts results in a full-length form of FGF5 and a short form that lacks the sequences encoded by exon 2 and most of exon 3 [1,6]. Functionally, it was demonstrated that the full-length FGF5 induces catagen, a function that is antagonized by the short form during anagen [5]. Indeed, the *angora* mouse mutant carries a 2-kb loss-of-function deletion in *Fgf5* and displays a delayed catagen onset and excessively long truncal hair [7]. Loss-of-function mutations associated with long-hair phenotypes have been described in several mammalian species including mice [4,8], dogs [9,10] and cats [11,12], which support a conserved role of FGF5 in the regulation of hair growth.

Among *Equidae*, the Poitou donkey breed (Baudet du Poitou) displays a long-hair coat. This breed originated from the Poitou region of France and was originally developed to produce a large type of working mules with the Poitevin Mulassier draft horse. The exact origin of the Poitou breed is unknown, probably dating back to before 1884 when the studbook was created in France. During the eighteenth and nineteenth centuries, thousands of Poitou mules were produced and exported to Europe. After World War II, draft animals were replaced by engines and mule production began to drop off. In 1977, the breed was endangered with only 44 Poitou donkeys (20 males and 24 females) registered in France. Thus, an efficient breeding program was set up to save the breed, which led to the reconstitution of a larger breeding stock. In 2005, 82 jacks and 355 jennets were used in breeding schemes (http://www.france-trait.fr).

Poitou is recognized as a large-sized donkey breed with a long-hair coat. When Poitou donkeys are left ungroomed, their coat retains very long hair that becomes matted and tangled and then grows down into a great coat. We hypothesized that the long-hair phenotype in the Poitou breed could result from loss-of-function mutations in the *FGF5* gene. Here, we report the precise identification of two loss-of-function alleles in *FGF5* that are fully associated with the long-hair phenotype in the panel of animals analysed when they are present as composite or homozygous recessive mutations.

## Methods

### Animals and ethics statement

One hundred and two donkeys from seven breeds were included in the study. They were all sampled in France from September 2012 to October 2013 and originated from Poitou (n = 35), Bourbonnais (n = 2), Cotentin (n = 10), Berry Black (n = 8), Normand (n = 18), Provence (n = 16) and Pyrenean (n = 13) breeds.

All the animals anlysed were included at the owner’s request. Pictures, buccal swabs and hair samples were sent directly by the owners or collected by a certified veterinarian (MA).

DNA samples from 131 horses and ponies previously collected for DNA identification purposes were used as controls. The panel included 40 thoroughbred, 33 French saddlebred, 26 Arab horses and eight Connemara, one Icelandic, seven Haflinger, seven French, three Pottok and six Shetland ponies. All animals were client-owned animals, which underwent no invasive sampling procedure, thus, no authorization for animal experiment was required according to the legal definition in Europe (Subject 5f of Article1, Chapter I of the Directive 2010/63/UE of the European Parliament and of the Council).

### DNA extraction

DNA was extracted from buccal swabs or hair roots using a Maxwell® 16 Instrument (Promega Corporation, Madison, USA), according to the manufacturer’s protocols.

### Sequencing and genotyping of *FGF5*

When this project was initiated, the donkey genome sequence was not yet released [13]. Thus, reference genome sequences were based on the equine genome sequence (Equine *FGF5* gene, [Ensembl:ENSECAG00000015710]). PCR and sequencing primers were designed using Primer3 [14]. Exon 1 was amplified using *FGF5* exon1 F 5′-AGCGCCGAGATCCGTTC-3′ and *FGF5* exon1 R 5′-GGACGGGTTTTGGAGGAG-3′ primers. Exon 2 was amplified using *FGF5* exon2F 5′-TGCAGTAATAAAGAATGGGAAG-3′ and *FGF5* exon2R 5′-TGCATTCCATTCTACAAACG-3′ primers. Exon 3 was amplified using *FGF5* exon3F 5′-GCCCATGGAATTCTTGGTTC-3′ and *FGF5* exon3R 5′-GCTGAAGCTGTGTCCAAAAGTG-3′ primers. A more detailed description of PCR and sequencing primers is provided in Additional file 1: Table S1. PCR amplicons were sequenced in both forward and reverse directions using the Sanger method (GATC Biotech AG, Konstanz, Germany), and electropherograms were manually inspected with Chromas Lite (Technelysium Pty Ltd, South Brisbane, Australia). Multiple alignments were performed using Multalin [15].

The genotype of the *FGF5* c.433_434delAT deletion was determined using a pyrosequencing method adapted from Ahmadian et al. [16] on a PyroMark Q96 ID pyrosequencer (Qiagen, Hilden, Germany) and the following forward and reverse PCR primers for *FGF5* c.433-434delAT: 5′-AATACGAGGAGTTTTCAGCAACAA-3′ and 5′-BIOTIN-CTTGCATGGAGTTTTCCTTTTT-3′, and the sequencing primer 5′-AGCAACAAATTTTTAGCG-3′.

The genotype of the *FGF5* c.245G > A SNP was determined using the same method and the following forward and reverse PCR primers :5′-TTGGAGCAGGGCAGTTTC-3′ and 5′-BIOTIN-TTGCCATCCGGGTAGATC -3′ and the sequencing primer 5′-CAGGGCAGTTTCCAG-3′. A more detailed description of genotyping primers is provided in Additional file 1: Table S2.

### Comparison of protein sequences and structure prediction

The Ensembl sequence of equine FGF5 was used [Ensembl:ENSECAP00000013338]. Multiple alignments were performed using Multalin [15]. The potential negative impact of missense mutations was assessed using the HumVar-trained version of PolyPhen-2. This version is designed to distinguish between mutations with drastic effects and other variations including abundant mildly deleterious alleles [17]. Because the 3D-structure of FGF5 has not been experimentally resolved, homology modeling and fold recognition were performed using the Phyre server [18] and human FGF1, FGF2, FGF9, FGF10 and FGF20 as templates.

### Accession numbers

Genomic coding sequences of *FGF5* from Berry Black (short-hair) and Poitou (long-hair) donkeys (*Equus asinus*) were submitted to GenBank. Accession numbers are [GenBank: KJ725176] for the wild type short-hair allele, [GenBank: KJ725177] for the c.433_434delAT allele and [GenBank: KJ725178] for the c.245G > A allele.

## Results

**Figure 1:**
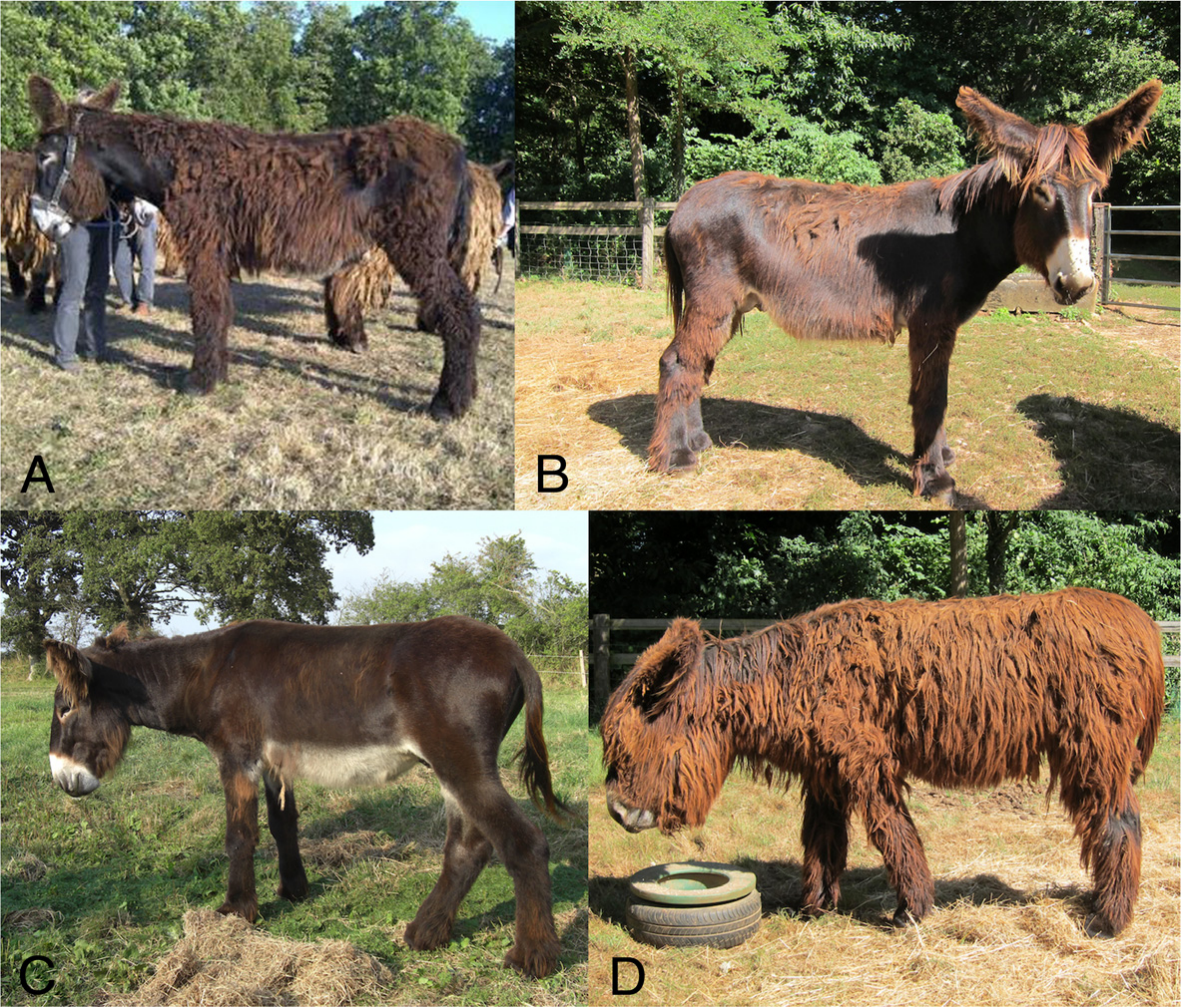
**Long-hair phenotype in Poitou donkeys.** Poitou donkeys exhibit lifelong hair growth with young animals usually showing a medium-sized coat with short-hair areas **(A, B, C)** and adult donkeys showing a long matted and tangled coat **(D)**.

The long-hair phenotype, which is very common in some domestic animals such as dogs and cats, is seldom observed in *Equidae* with the exception of the French Poitou donkey breed. Poitou donkeys are characterized by their large size and shaggy coat that is caused by long, soft hair arranged in long cords when ungroomed (Figure 1). Segregation analysis showed that long-hair is a recessive trait in donkeys. Indeed, F1 Poitou crossbred donkeys and mules are always short-haired. With the aim of providing breeders with an accurate DNA-based test to detect long-hair carriers in Poitou crossbred descendants, we performed a mutation screening of *FGF5* in various donkey breeds. Apart from comparative cytogenetic maps, whole-genome mapping tools are still lacking for this species [19,20]. Indeed, the SNP (single nucleotide polymorphism) array that was developed for the horse genome [21,22] is of little use for domestic donkeys, with a cross-species validation rate of 5.6 × 10^−3^ for the 53k SNP array [22]. Thus, to circumvent these limitations, we screened long-haired donkeys for mutations in the *FGF5* gene, which is the most relevant candidate gene to explain this variation in coat length. To cover the three exons and their respective intron-exon flanking sequences, we designed three sets of intron primers using the equine *FGF5* Ensembl sequence.

First, a panel composed of three long-haired Poitou donkeys and two short-haired Berry Black control donkeys was used. The three sets of primers yielded amplicons at the right size, and sequencing confirmed that *FGF5* sequences were successfully amplified. Global alignement revealed 99.7% similarity between the equine and donkey *FGF5* coding sequences, which confirmed that the two genes are orthologous. Furthermore, pair-wise base-to-base comparisons of sequences between donkeys and the horse reference sequence revealed two interspecific variants, one SNP and a two-base pair deletion from the individuals of this first panel (Table 1). Careful analysis of the two interspecific variants revealed that they consisted of a synonymous mutation and a non-synonymous mutation, which based on PolyPhen-2 prediction has no impact on FGF5 function (L223S; score 0.00). On the contrary, the donkey SNP is a nonsense mutation (c.245G > A) that leads to a stop codon at position 82 in FGF5 and the two-base pair deletion (c.433_434delAT) produces a frameshift that results in a stop codon at position 159 in FGF5 (Table 1).

**Table 1:** ***FGF5*** **polymorphisms in donkey exon sequences compared to the horse reference coding sequence**

	**c.245G > A**	**c.433_434delAT**	**c.546C > T**	**c.668T > C**
	**exon 1**	**exon 2**	**exon 3**	**exon 3**
Ensembl equine sequence	G	N	C	T
Poitou 1	*G/G*	*del/del*	*T/T*	*C/C*
Poitou 2	*G/G*	*del/del*	*T/T*	*C/C*
Poitou 3	*G/G*	*del/del*	*T/T*	*C/C*
Berry Black 1	*G/G*	*N/N*	*T/T*	*C/C*
Berry Black 2	*G/A*	*N/N*	*T/T*	*C/C*
Consequence	p.W82*	p.M145Vfs*15	p.(=)	p.L223S
PolyPhen-2 prediction				Benign

N: no deletion. *del*: AT deletion.

In a second series of experiments, the complete cohort composed of 102 donkeys representing seven breeds was genotyped for the c.245G > A SNP and the c.433_434delAT deletion. Among the 35 long-haired Poitou donkeys, 31 were homozygous for the *FGF5* mutated allele containing the c.433_434delAT deletion, while four Poitou, two Pyrenean, one Normand and one Bourbonnais donkeys were heterozygous. The remaining 63 short-haired donkeys were homozygous for the wild type reference allele (Table 2). Thus, we genotyped 131 short-haired ponies and horses from nine breeds but did not identify the deletion in this panel (Table 2), which supports the association (chi-square test, p = 2.62 × 10^−20^) between the Poitou deletion and the long-haired phenotype of this breed.

**Table 2:** **Genotypes of donkeys and horses for the c.433_434delAT deletion**

	***N/N***	***N/del***	***del/del***	**Total**
Pyrenean donkeys	11	**2**	0	13
Provence donkeys	16	0	0	16
Normand donkeys	17	**1**	0	18
Berry Black donkeys	8	0	0	8
Cotentin donkeys	10	0	0	10
Bourbonnais donkeys	1	**1**	0	2
Poitou donkeys	0	4*	**31**	35
***Total in donkeys***	63	**8**	**31**	102
Horses and ponies from nine breeds	131	0	0	131

*These four Poitou donkeys are the same animals as those shown in italics in Table 3; *N*: no deletion; *del*: AT deletion. Heterozygous and homozygous mutant animals are bolded.

To check whether the four Poitou individuals that were identified as heterozygous for the deletion were compound heterozygotes, we determined the genotypes of the cohort at the c.245G > A SNP. Indeed the four c.433_434delAT heterozygous Poitou individuals were also heterozygous for the c.245G > A nonsense mutation (Table 3). Ninety-three donkeys were homozygous for the wild type reference allele, and interestingly the five remaining donkeys from the cohort (one Berry Black, one Normand, one Pyrenean and two Provence) carried a copy of the mutant allele (Table 3).

**Table 3:** **Genotypes of donkeys for the c.245G > A SNP**

	***G/G***	***G/A***	***A/A***	**Total**
Pyrenean donkeys	12	**1**	0	13
Provence donkeys	14	**2**	0	16
Normand donkeys	17	**1**	0	18
Berry Black donkeys	7	**1**	0	8
Cotentin donkeys	10	0	0	10
Bourbonnais donkeys	2	0	0	2
Poitou donkeys	31	4*	0	35
**Total**	93	9	0	102

*These four Poitou donkeys are the same animals as those shown in italics in Table 2. Heterozygous animals are bolded.

**Figure 2:**
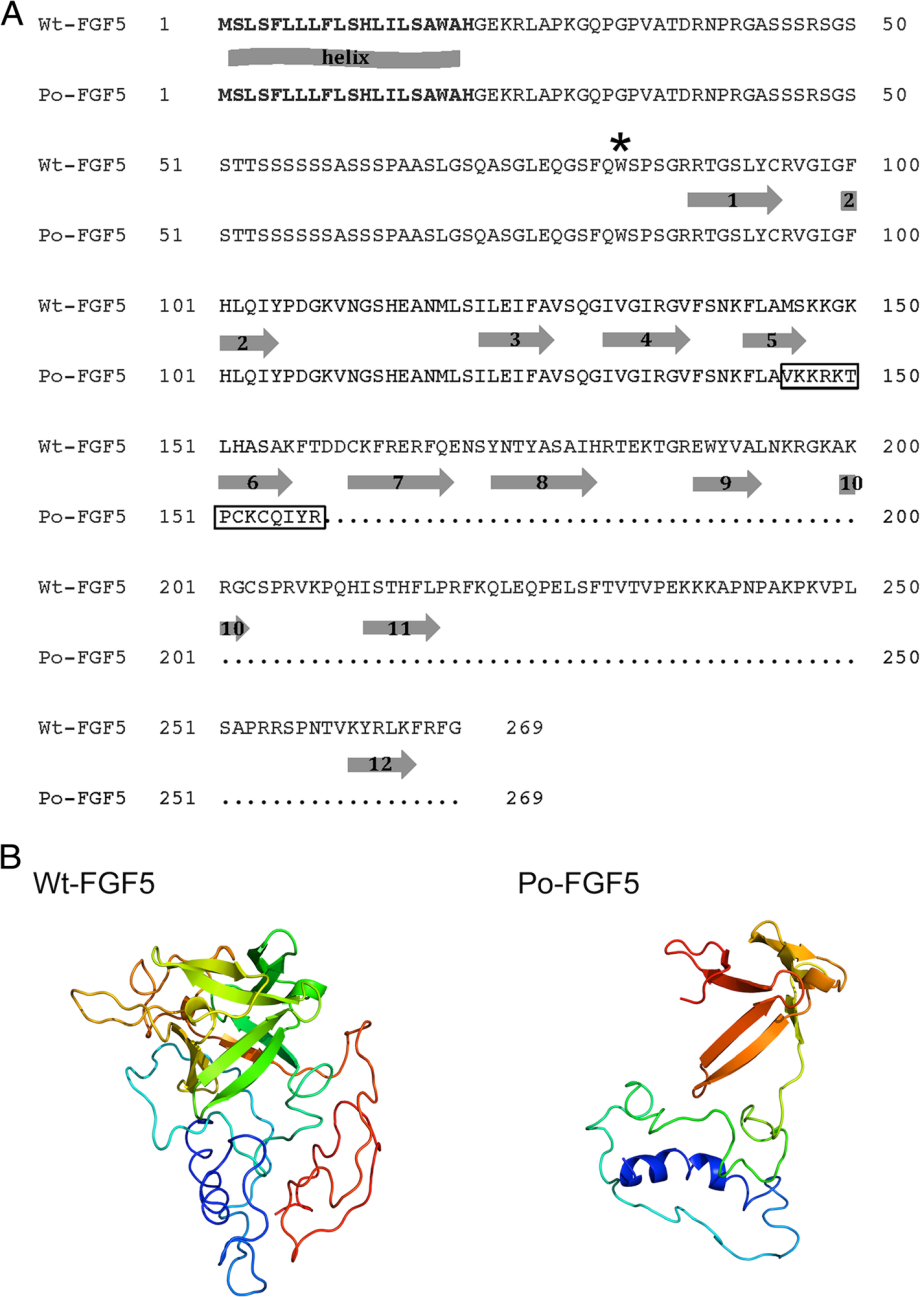
**Wild type and mutant FGF5 proteins. (A)** Alignment of the FGF5 amino-acid sequence predicted from the equine wild type allele (Wt-FGF5, [Ensembl:ENSECAT00000016512]) and the c.433_434delAT mutant Poitou allele (Po-FGF5). The signal peptide is indicated in bold characters. The secondary structure predicted by the Phyre^2^ program is represented by the arrows below the Wt-FGF5 sequence. Arrows with numbers represent the 12 β-strands. The predicted N-ter α-helix is indicated. The frameshift produced by the c.433_434delAT deletion is framed. A star indicates the location of the stop codon produced by the c.245G > A nonsense mutation. **(B)** 3D models built using the Phyre server represent the horse wild type Wt-FGF5 (left) and the donkey c.433_434delAT Poitou Po-FGF5 (right) proteins; the α-helix is highlighted in blue and the β strands are depicted by arrows.

As stated above, the c.433_434delAT deletion induces a frameshift that leads to a premature stop codon at position 159 in the protein. To evaluate the putative functional impact of the c.433_434delAT deletion on FGF5, 3D models were built using the Phyre server [18] and five human FGF proteins as templates. The models predicted that the truncated protein lacked seven of the 12 β-strands that define the canonical FGF trefoil (Figure 2; [23]), which supports the hypothesis that the c.433_434delAT deletion can impair FGF5 function. Similarly, the models predicted a loss-of-function for the shorter, truncated protein produced from the c.245G > A allele and which lacked all β-strands (Figure 2).

To conclude, we propose that the prevalent c.433_434delAT deletion identified in all Poitou donkeys constitutes the recessive long-hair *FGF5*^*l-poitou*^ Poitou allele and that the c.245G > A nonsense mutation, which is rarely identified in the Poitou and other breeds, constitutes a long-hair *FGF5*^*l-donkey*^ donkey allele.

## Discussion

Although the long-hair phenotype is a distinctive characteristic of the Poitou donkey breed, the molecular mechanisms responsible for this trait have not yet been reported. Since the mapping tools that have been developed for horses are poorly efficient in donkeys [22], we opted for a candidate gene approach that targeted the *FGF5* gene for which loss-of-function mutations have been shown to produce a conserved long-hair phenotype in mammals (http://omia.angis.org.au). Due to the high conservation between donkey sequences and the horse reference genome, we successfully amplified the complete *FGF5* coding sequence in donkeys using intron primers that were designed from the horse sequence [21]. Among the polymorphisms identified, the Poitou-specific c.433_434delAT deletion was postulated to be a deleterious mutation, since the resulting allele produces a truncated protein that lacks seven of the 12 β-strands involved in FGF5 function. Indeed, if this allele is present in two copies, it is associated with the long-hair phenotype in ~90% (31/35) of the Poitou donkeys from our cohort. The remaining 10% of long-haired Poitou donkeys from the cohort could be explained by the combined inheritance of this c.433_434delAT deletion with another recessive c.245G > A mutation. Interestingly, the latter mutation was also detected as a single copy in individuals from four other short-hair donkey breeds.

We observed high levels of similarities between the donkey FGF5 protein sequence and several mammalian orthologs, which emphasizes that the wild type FGF5 protein may also be functional in donkeys. FGF ligands carry out their diverse functions by binding and activating FGF receptors (FGFR), which belong to the family of tyrosine kinase receptors, in a heparan sulphate glycosaminoglycan (HSGAG)-dependent manner [2,23,24]. Thus, binding of the FGF ligand to HSGAG and FGFR promotes dimerization and stabilization of the FGF-HSGAG-FGFR complex, which induces the activation of tyrosine kinase and the downstream intracellular signaling processes [24]. While the HSGAG binding site of FGF is composed of the β1-β2 loop and parts of the region spanning β10 and β12 [2], residues involved in the FGFR interaction are located in several regions including β1, β2, β9 and β12 strands, β1-β2 and β8-β9 turns, β3-β4 loop for the FGF - D2 region of FGFR and in regions including β1 and β4 to β8 sheets and β4-β5, β7-β8 and β8-β9 loops for the FGF - D3 region of FGFR [23].

It should be noted that the 12 β-strands and critical residues involved in the binding of FGF5 with HSGAG and FGFR interactions are conserved in horses and donkeys (Figure 2 and [23]), which suggests that the wild type FGF5 protein is functional in both species. In silico, the c.245G > A and the c.433_434delAT mutant alleles yield truncated FGF5 proteins that lack all or the last seven β-strands, respectively (Figure 2). If correctly addressed within the cell and secreted, the encoded truncated proteins are thus unlikely to mediate the FGF5 signaling process, which strongly suggests that *FGF5*^*l-poitou*^ and *FGF5*^*l-donkey*^ are two loss-of-function donkey alleles of *FGF5*. This is in agreement with long-haired phenotypes previously observed in FGF5-deficient mice, humans, dogs and cats [4,7-12].

To the best of our knowledge, long-haired horses have never been reported. In contrast, four long-haired donkey breeds have been described, including the Poitou breed. For this breed, we provide strong evidence that all individuals are deficient in FGF5 signaling, either because they carry two copies of the most prevalent loss-of-function *FGF5*^*l-poitou*^ allele, or because they are compound heterozygotes carrying the two loss-of-function *FGF5*^*l-poitou*^ and *FGF5*^*l-donkey*^ alleles. Interestingly, these two alleles were also detected at low frequency in heterozygous individuals from short-haired French breeds. The probability that the two *FGF5*^*l-poitou*^ and *FGF5*^*l-donkey*^ alleles could have independently arisen in several breeds as a consequence of distinct mutation events is very low. It is more likely that they result from founder mutations that were disseminated because of outcrossing. Altough the genealogy of the donkeys included in this study is not known, the hypothesis of common ancestors is supported by the history of French breeds. Indeed, the studbooks of Berry Black, Normand and Bourbonnais breeds are very recent, i.e. they were created in 1994, 1997 and 2002, respectively. In 2011, 34 Berry Black and 66 Normand donkeys were registered in France, and in 2012, 10 Bourbonnais donkeys were registered (http://www.haras-nationaux.fr). Previous registrations were accepted based on aesthetic criteria, which has certainly favored individuals with genomic admixture of other breeds.

Other long-haired donkeys belong to the American miniature donkey breed that displays the so-called “woolly phenotype”, and to the two endangered, related Spanish Zamorano-Leonés and Portuguese Burro de Miranda breeds. In the 1990s’, Zamorano jennies were imported to the French Poitou region during a preservation program, and, in the last decades, many Spanish donkeys were imported to France. Thus, it can be hypothesized that the *FGF5*^*l-donkey*^ allele was introgressed from the Spanish breeding stock, which is supported by genealogical analyses that revealed that the four Poitou compound heterozygotes of our cohort were descendants of Zamorano jennies. Moreover, it is highly problable that the most prevalent *FGF5*^*l-poitou*^ allele was fixed by Poitou breeders during the 19^th^ century, which corresponded to the golden period of this breed. Haplotype analyses on a larger cohort of donkeys, including Miranda and Zamorano individuals, would help confirm these hypotheses. Currently, the small number of registered donkeys in each breed prevents more in-depth analyses.

## Conclusions

Our results highlight the allelic heterogeneity of the long-hair phenotype in donkeys and enlarge the panel of recessive *FGF5* loss-of-function alleles described in mammals. Identification of mutations that underlie the long-hair phenotype in donkeys will help breeders to detect heterozygous carriers in short-haired French breeds and thus prevent matings at risk to produce non-eligible long-haired foals. Finally, it would be interesting to test for the *FGF5*^*l-poitou*^ and *FGF5*^*l-donkey*^ alleles the American miniature donkeys that have been reported to be woolly-haired and that were produced in the USA. The DNA test developed from this study is available to the community and will help breeders to identify carriers in their breeding stocks.

## Additional file

Additional file 1: Table S1. PCR and sequencing primers. The data provided represent the sequences and annealing temperatures for the primers that were used to amplify and sequence the three *FGF5* exons. **Table S2.** Genotyping primers. The data provided represent the two sets of three primers that were used to amplify and pyrosequence the two *FGF5* mutations identified in donkeys.
