# Supplementary material for: Two recessive mutations in FGF5 are associated with the long-hair phenotype in donkeys
Source: Genet Sel Evol. 2014 Sep 25;46(1):65. doi: 10.1186/s12711-014-0065-5 (PMC4175617; doi:10.1186/s12711-014-0065-5)
Supplement: Additional file 1: Table S1. — PCR and sequencing primers. The data provided represent the sequences and annealing temperatures for the primers that were used to amplify and sequence the three FGF5 exons. Table S2. Genotyping primers. The data provided represent the two sets of three primers that were used to amplify and pyrosequence the two FGF5 mutations identified in donkeys. [file 12711_2014_65_MOESM1_ESM.docx]

Additional file 1

**Table S1: PCR and sequencing primers**

| Exon | **Forward primer** | **Forward primer sequence** | **Tm** | **Reverse primer** | **Reverse primer sequence** | **Tm** | **PCR product** |
| --- | --- | --- | --- | --- | --- | --- | --- |
| *FGF5*exon1 | *FGF5*exon1F | agcgccgagatccgttc | 58°C | *FGF5*exon1R | ggacgggttttggaggag | 58°C | 568bp |
| *FGF5*exon2 | *FGF5*exon2F | tgcagtaataaagaatgggaag | 54°C | *FGF5*exon2R | tgcattccattctacaaacg | 53°C | 271bp |
| *FGF5*exon3 | *FGF5*exon3F | gcccatggaattcttggttc | 57°C | *FGF5*exon3R | gctgaagctgtgtccaaaagtg | 60°C | 551bp |

**Table S2: Genotyping primers**

| **Mutation** | **Primer** | **Sequence** | **Tm** | **PCR product** |
| --- | --- | --- | --- | --- |
| c.433_434delAT | Forward | AATACGAGGAGTTTTCAGCAACAA | 55°C | 63bp |
|  | Reverse | BIOTIN-CTTGCATGGAGTTTTCCTTTTT | 57°C |  |
|  | Sequencing | AGCAACAAATTTTTAGCG | 47°C |  |
| c.245G>A | Forward | TTGGAGCAGGGCAGTTTC | 56°C | 104bp |
|  | Reverse | BIOTIN-TTGCCATCCGGGTAGATC | 56°C |  |
|  | Sequencing | CAGGGCAGTTTCCAG | 50°C |  |
